# Baicalein is an available anti-atherosclerotic compound through modulation of nitric oxide-related mechanism under oxLDL exposure

**DOI:** 10.18632/oncotarget.10263

**Published:** 2016-06-23

**Authors:** Shih-Hung Chan, Ching-Hsia Hung, Jhih-Yuan Shih, Pei-Ming Chu, Yung-Hsin Cheng, Yi-Ju Tsai, Huei-Chen Lin, Kun-Ling Tsai

**Affiliations:** ^1^ Department of Internal Medicine, National Cheng Kung University Hospital, College of Medicine, National Cheng Kung University, Tainan, Taiwan; ^2^ Institute of Allied Health Sciences, College of Medicine, National Cheng Kung University, Tainan, Taiwan; ^3^ Department of Physical Therapy, College of Medicine, National Cheng Kung University, Tainan, Taiwan; ^4^ Department of Internal Medicine, Chi-Mei Hospital, Tainan, Taiwan; ^5^ Department of Anatomy, School of Medicine, China Medical University, Taichung, Taiwan; ^6^ Department of Education and Research, Taipei City Hospital, Taipei, Taiwan; ^7^ Department of Physical Therapy, Shu-Zen Junior College of Medicine and Management, Kaohsiung, Taiwan

**Keywords:** oxidized low-density lipoprotein, endothelial cells, baicalein, nitric oxide, reactive oxygen species, Gerotarget

## Abstract

OxLDL facilitate reactive oxygen species (ROS) formation and up-regulation of the executioner caspase-3 via the mitochondrial apoptotic pathway involves several critical steps in human endothelial cells. Previous studies reported that oxLDL-facilitated endothelial oxidative stress is associated with impairment of eNOS and up-regulation of inducible nitric oxide synthase (iNOS). Baicalein is the most abundant component that has anti-HIV, anti-tumor, anti-oxidant and free radical scavenging functions. In this present study, we shown that baicalein hinibits oxLDL-caused endothelial dysfunction through suppression of endothelial inflammation and oxidative stress that causes to cellular apoptosis. Specifically, baicalein reduces the elevation of ROS concentration, which subsequently inhibits the oxLDL-decreased expression of anti-oxidant enzymes, enriches the bioavailability of NO, stabilizes the mitochondrial membrane, thereby inhibiting the discharge of cytochrome c from mitochondria, a molecule required for the activation of the pro-apoptotic protein caspase 3. However, inhibition of eNOS impairs the anti-apoptotic and anti-inflammatory effects of baicalein. These results provide new insight into the possible molecular mechanisms by which baicalein protects against atherogenesis by NO-related pathways.

## INTRODUCTION

Oxidized low-density lipoprotein (oxLDL) are cytotoxic and cause dysfunction and cell death in cultured vascular cells. The morphological alterations of endothelial cells associated with oxLDL toxicity are similar to those found *in vivo* on endothelial cells covering atherosclerotic lesions [1]. OxLDL induce the endothelial cell generation of ROS and activation of the executioner caspase-3 *via* the mitochondrial apoptotic mechanisms involves several critical steps [2]. A previous study indicates that oxLDL stimulate expression of the pro-apoptotic protein tumor suppressor p53 which contributes to apoptosis in endothelial progenitor cells by up-regulating Bax and downregulating Bcl-2 expression [3]. Bcl-2 family members regulate many apoptosis-related functions of the mitochondria. Anti-apoptotic Bcl-2 homologues such as Bcl-2, Bcl-xL, and A1, mitigate the depolarization of membrane and the discharge of cytochrome c from mitochondria. Pro-apoptotic homologues, such as trBid, Bax, as well as Bak, can impair the ability of Bcl-2 [4]. Bax and Bid may insert into the mitochondrial membrane elevate the permeability of the membrane and cause to cytochrome c release [5]. After that, cytochrome c associating with Apaf-1 combine with caspase-9. In this complex cytochrome c acts as a co-factor for activating caspase-9 [6]. Then cleaves pro-caspase-3, activation of caspase-3, which plays as a killer, through cleaving multiple of another substrate within the cells, result in inducing a large chromatin condensation and DNA fragmentation [7].

Oxidative stress is caused by reactive oxygen species (ROS) are molecules containing unpaired electrons, which is derived from many cellular enzyme systems within the cardiovascular system [8]. Many pathological cardiovascular diseases are associated with increased production of ROS in vascular tissues, including hypertension, hyperlipidemia, and diabetes [9]. Elevated oxidative stress acts a critical role in endothelial dysfunction and atherogenesis [10]. Endothelial cells generate ROS, involving nitric oxide (NO), peroxynitrite (.ONOO-), superoxide (O2-.), hydrogen peroxide (H2O2), hydroxyl radicals (.OH), and other radicals. The potential enzymatic sources of endothelial superoxide anion include mitochondria, xanthine oxidase, uncoupled NO synthases, lipoxygenase, cytochrome P450 enzymes, and nicotinamide adenine dinucleotide phosphate (NADPH) oxidase [11].

Previous studies suggested that oxLDL-caused endothelial oxidative damage is associated with impairment of eNOS and up-regulation of inducible nitric oxide synthase (iNOS). Reactive oxygen species (ROS), especially superoxide, formated by oxLDL directly responds with NO to form peroxynitrite, a stable molecule that is toxic to endothelial cells. As a scavenger of free radical, NO mitigates the production of hydrogen peroxide and hinders the activation of NF-κB and the subsequent expression of inflammatory events that promote leukocyte adhesion [12] and macrophage recruitment [13].

Baicalein, an of natural phenolic anti-oxidant isolated from Scutellaria baicalensis (S. baicalensis) Georgi (Huangqinin Chinese). S. baicalensis Georgi contains many kinds of flavones, phenylethanoids, amino acids, sterols and essential oils. Its dried roots contain over 30 kinds of flavonoids. Baicalein is the most abundant component that has anti-oxidant and free radical scavenging effects [14, 15]. In addition, report has shown that baicalein can scavenge ROS generation during hypoxia, simulate ischemia-reperfusion in a chick cardiomyocyte and protect against cell death [16]. The benefits of baicalein on vascular diseases showing that baicalein has positive effects on attenuation of intercellular adhesion molecule-1 (ICAM-1) expression in cultured human endothelial cells induced by interleukin 1β and tumor necrosis factor α [17].

Although baicalein has been shown to have anti-oxidant effects both *in vivo* and *in vitro*, to the best of our knowledge, there are no studies of these effects on oxLDL-induced endothelial dysfunction. Therefore, the aim of this study was to examine whether baicalein could protect against oxLDL-induced endothelial dysfunction and explore the possible mechanisms. We undertook the current study to explore the direct effects of baicalein on oxLDL-induced several apoptotic features, such as mitochondrial destabilization, cytochrome c release and the activation of caspase-3. We also we explored whether baicalein protects against attenuates oxLDL-induced damage by modulating the NO-related pathways.

## RESULTS

### Baicalein decreased DNA damage and cell death induced by oxLDL in endothelial cells

**Figure 1:**
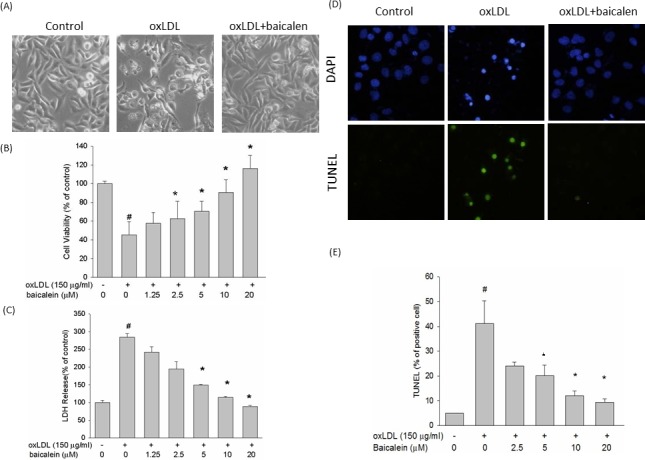
Effects of baicalein on oxLDL-induced endothelial cell death HUVECs were incubated with oxLDL (150 μg/ml) in the absence or presence of indicated concentrations of baicalein. Photomicrographs from phase-contrast microscopy **A.** Viability was determined *via* MTT assay **B.** and LDH release **C.** HUVECs were incubated with oxLDL in the absence (middle) or presence (right) of baicalein for 24 h. Late apoptotic death of oxLDL-exposed HUVECs was evaluated using the TUNEL assay **D.**, **E.** The values represent means±SEM from three separate experiments. #*P* < 0.05 *vs*. control; *P < 0.05 *vs*. oxLDL treatment.

Phase-contrast microscopy was performed to examine the protective effects of baicalein on morphological features of HUVECs after exposure to oxLDL. After a 24 hrs exposure to oxLDL, the number of shrunken cells or cells with blebbing membranes was significantly reduced by the presence of baicalein (Figure 1A). The viability of cells incubated with oxLDL in the absence or presence of indicated concentrations of baicalein was assessed using the MTT assay (Figure 1B), and membrane permeability was assayed by LDH release (Figure 1C). Our results showed that oxLDL significantly reduced viability and increased membrane permeability in HUVECs after 24 hrs of incubation; however, pretreatment oxLDL-induced cytotoxicity of endothelial cells dose dependently. The TUNEL and DAPI staining assays were then used to clarify the protective effects of baicalein against oxLDL-induced DNA damage. As shown in Figure 1D, 1E, cells incubated with oxLDL for 24 hrs showed typical features of apoptosis, including the formation of condensed nuclei. Those morphologic features were not observed in endothelial cells pretreated with baicalein.

### Baicalein mitigated oxLDL-caused intracellular ROS generation in endothelial cells

**Figure 2:**
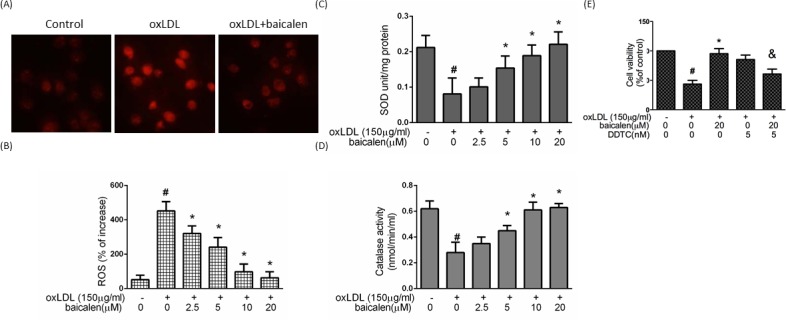
The protective effects of baicalein on oxLDL-mediated ROS generation in HUVECs After preincubation for 2 hrs with the indicated concentrations of baicalein (2.5-20 μM), HUVECs were incubated with the DHE for 1 hr, followed by treatment with oxLDL. **A.** Fluorescence images exhibited the ROS level in control cells (left) and HUVECs stimulated with oxLDL (middle) in the presence of baicalein (right). **B.** Fluorescence intensity of HUVECs was measured with a fluorescence microplate reader. Fluorescence distribution of DHE oxidation was expressed as a percentage of increased intensity. The activity of **C.** SOD and **D.** catalase in HUVECs stimulated with oxLDL in the absence or presence of indicated concentrations of baicalein were determined. **E.** HUVECs were incubated with oxLDL (150 μg/ml) in the absence or presence of baicalein. In some groups, SOD inhibitor (DDTC) was treated before expose to oxLDL and baicalein. Viability was determined *via* MTT assay. Data are expressed as the mean±S.E. of three independent analyses. #*P* < .05 *vs*. untreated control; *P < 0.05 *vs*. oxLDL treatment. &*P* < .05 *vs*. oxLDL+ baicalein treatment.

To understand whether the observed anti-apoptotic effect of baicalein can be attributed to inhibition of ROS generation. We confirmed that treatment with oxLDL for 2 hrs generated a seven-fold enlargement in ROS formation. Pretreatment of endothelial cells with baicalein led to an inhibition in ROS (Figure 2A, 2B). To test the mechanisms comprised in the anti-oxidant function of baicalein in endothelial cells under oxLDL stimulation, we investigated the activities of the anti-oxidant enzyme in endothelial cells exposed with oxLDL. In Figure 2C, 2D, the activity of SOD and catalase were decreased in oxLDL-treated endothelial cells, however, the intervention of cells with baicalein obviously maintained the activity of those anti-oxidant enzymes. In addition, we found the SOD inhibitor (diethyldithiocarbamate ; DDTC) impaired the cyto-protective effect of baicalein, indicating that baicalein protects against oxLDL-caused endothelial death by modulation of antioxidant enzymes (Figure 2E).

### Baicalein reduced oxLDL-facilitated eNOS down-regulation and iNOS, nitrotyrosine up-regulation

**Figure 3:**
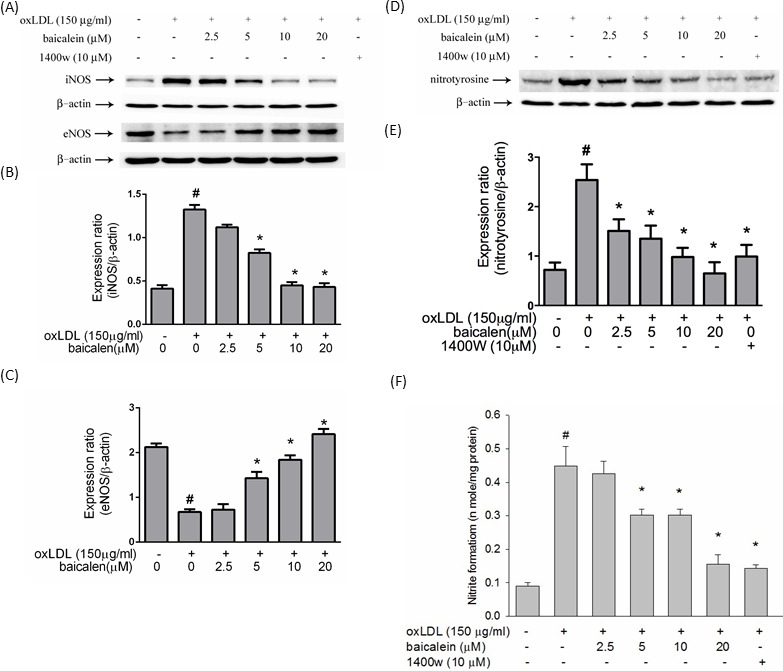
Effects of baicalein on oxLDL up-regulated iNOS, down-regulated eNOS, nitrotyrosine protein expression and oxLDL-enhanced nitrite accumulation The protein expression of iNOS, eNOS **A.** and nitrotyrosine **D.** were analyzed by Western blot pretreated with baicalein (20 μM) for 2 hours followed by oxLDL(150 μg/ml) for 24 hours in HUVECs. Anti-β-actin antibody was used for normalization of cytosolic proteins. **E.** Content of NO was assayed using Griess reagent. Data of bar figure represent mean±SEM of 3 independent analyses. # *P* < 0.05 compared with control and *P < 0.05 compared with oxLDL-stimulated HUVECs.

ROS, especially superoxide, produced by oxLDL directly reacts with NO to form peroxynitrite, a stable molecule that is toxic to endothelial cells. In Figure 3A, 3B, 3C, oxLDL impaired eNOS expression levels and increased iNOS protein expression levels reversed to levels close to those seen in control cells when cells were treated with baicalein prior to exposure with oxLDL. Furthermore, the oxLDL-enhanced nitrotyrosine of tyrosine and nitrite (NO2-) accumulation were suppressed in HUVECs pretreated with baicalein as well as they were also inhibited by iNOS-specific inhibitor 1400W (Figure 3D, 3E, 3F).

### Baicalein-mediated anti-inflammatory ability entailing eNOS function

**Figure 4:**
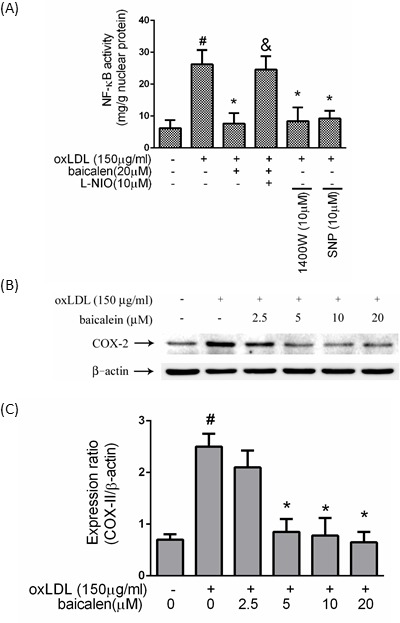
Effects of baicalein on oxLDL-induced NF-κB activation HUVECs were pretreated with each inhibitor 1 hr before incubated with oxLDL. Nucleic proteins were extracted for nuclear translocation assay of NF-κBp65 **A.** Effects of baicalein on COX-2 levels were assessed by Western blotting. **B.**, **C.** The values represent means±S.E. from three separate experiments. #*P* < .05 *vs*. untreated control; *P < 0.05 *vs*. oxLDL treatment. &*P* < .05 *vs*. oxLDL+ baicalein treatment.

We hypothesized that oxLDL causes NF-κB activation by inhibiting the bioavailability of NO and that oxLDL-caused NF-κB activation could be returned by baicalein intervention. In Figure 4A, intervention of endothelial cells with baicalein conspicuously reduced the oxLDL-caused activation of NK-κB. In addition, HUVECs pretreatment with L-NIO impaired the protective ability of baicalein. Cells pretreated with 1400W or exogenous donor of NO (SNP) revealed a significant mitigation in the activation of NF-κB. COX-2 was mediated by NF-κB. Our results suggested that pretreatment with baicalein mitigated the expression levels of COX-2 (Figure 4B, 4C).

### Baicalein attenuated oxLDL-induced pro-apoptotic events

**Figure 5:**
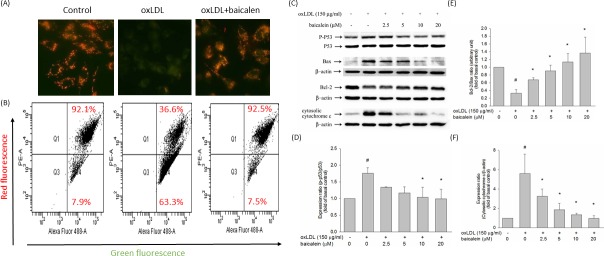
Effect of baicalein on mitochondrial transmembrane permeability transition and apoptosis Cells were incubated with 20 μM baicalein for 2 hours and then incubated with 150 μg/ml oxLDL for an additional 24 hours. **A.** The change in mitochondrial membrane potential was assessed based on the signal intensity from monomeric and J-aggregate JC-1 fluorescence as described in materials and methods. (left) No treatment; (middle) oxLDL; and (right) oxLDL + baicalein. **B.** JC-1 fluorescence was confirmed by flow cytometry. Green fluorescence intensity indicates the cells with low ΔΨm, while the red fluorescence intensity indicated the cells with stable ΔΨm. Effects of baicalein on dephosphorylation of p53, analysis of Bcl-2 family proteins, and mitochondrial cytochrome c release in response to oxLDL and baicalein. HUVECs were incubated with 150 mg/ml oxLDL in the absence or presence of indicated concentrations of baicalein for 24 hrs. Representative, Western blots **C.** and summary data **D.**,**E.**,**F.** show that oxLDL upregulated pro-apoptotic (Bax) proteins and downregulated anti-apoptotic (Bcl-2) proteins and increased the abundance of cytochrome c in the cytosolic fraction, whereas baicalein pretreatment suppressed these apoptosis-provoking alterations. Results were subjected to densitometric analysis. Data of bar figure represent means±SEM of 3 independent analyses. # *P* < 0.05 compared with control and *P < 0.05 compared with oxLDL-stimulated HUVECs.

To examine whether reduction of mitochondrial disruption accounts for the anti-apoptotic effect of baicalein, we investigated the effect of oxLDL on mitochondrial permeability. When endothelial cells were stimulated to oxLDL, the ΔΨm was depolarized, as shown by the increase in green fluorescence. Pretreatment with baicalein reduced the change in ΔΨ m, as indicated by repression of green fluorescence and restoration of red fluorescence (Figure 5A). Quantitative analysis from flow cytometry supported these findings (Figure 5B). In human endothelial cells, oxLDL-caused oxidative stress leads to the activation of P53, which subsequently induces a conformational change in Bax that enables the mitochondrial translocation of that proapoptotic protein. Our results revealed that baicalein significantly mitigated the activation of P53 and the expression of Bax and significantly enriched the expression of the anti-apoptotic protein Bcl-2(Figure 5C, 5D, 5E). It is known that disruption of mitochondrial membrane function results in the release of the mitochondrial enzyme cytochrome c into the cytosol. Therefore, mitochondria were separated from the cytosolic fraction and detected by Western blotting. As shown in Figure 5F, the amount of cytochrome c released into the cytosolic fraction was much greater in endothelial cells that had been incubated with oxLDL than in control cells and baicalein mitigated the cytosolic cytochrome c levels in oxLDL-exposed endothelial cells.

### Baicalein attenuated oxLDL-activated caspase 3 expression

**Figure 6:**
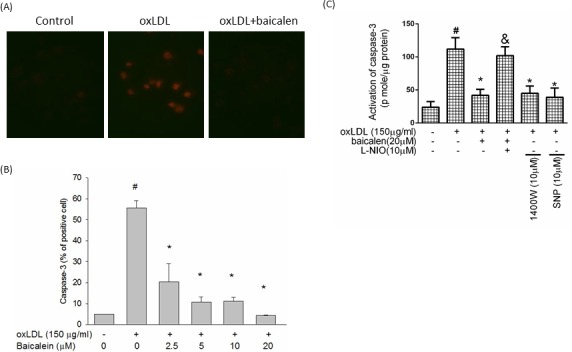
Effects of baicalein on oxLDL-induced caspase 3 activation **A.** HUVECs were incubated for 1 hr with indicated concentrations of baicalein, followed by exposure to oxLDL for another 24 hrs. Fluorescent images show the activated caspase 3 level in control cells (left), HUVECs stimulated with oxLDL (middle), and in the presence of baicalein (right). **B.** Fluorescence intensity of cells was measured by flow cytometry. **C.**The activity of caspase 3 was measured by EnzCaspase-3 assay kit. Data are expressed as the mean±S.E. of three independent analyses. #*P* < .05 *vs*. untreated control; *P < .05 *vs*. oxLDL treatment. &*P* < .05 *vs*. oxLDL+ baicalein treatment.

Next, we tested the effects of baicalein on oxLDL-induced activation of caspase 3 using fluorescence microscopy and flow cytometry. As shown in Figure 6A and 6B, baicalein attenuated oxLDL-induced caspase 3 activation. We also determined the activity of caspase 3 using the EnzCaspase-3 assay kit. As revealed in Figure 6C, baicalein attenuated the cleavage of caspase 3 activated by oxLDL. Pretreatment of cells with baicalein attenuated and eNOS inhibitor partially abolished the inhibitory effects of baicalein on caspase 3 activity. Moreover, the addition of 1400W or SNP definitely reduced oxLDL-activated caspase 3 expression.

## DISCUSSION

In this present study, we revealed that baicalein protected against oxLDL-caused endothelial dysfunction by repressing endothelial inflammation and oxidative stress that causes to cellular apoptosis. Specifically, baicalein mitigated the formation of ROS, which subsequently repressed the oxLDL-decreased expression of anti-oxidant enzymes, promoted the bioavailability of NO, stabilized the mitochondrial membrane, thereby repressing the release of cytochrome c, a molecule required for the activation of the pro-apoptotic protein caspase 3.

Atherosclerotic regions have reduced the activity of eNOS and reduction of locally released NO may enhance oxidative stress and cell proliferation. In contrast, the level of NO produced by inducible NO synthase (iNOS) is several orders of magnitude higher than that produced by eNOS in atherosclerotic lesions [18]. In addition, the increase in NADPH oxidase activity leads to eNOS uncoupling, which results in the generation of superoxide rather than NO and it plays an important role in the regulation of vascular tone [19]. In the present study showed nitrite formation was largely parallel to the expression level of iNOS (Figure 3), suggesting that generation of NO as a precursor of nitrite in oxLDL-treated cells is due mainly to iNOS. However, pretreatment of HUVECs with baicalein suppressed the oxLDL-induced up-regulation of iNOS, thereby leading to a reduction in protein nitrotyrosine (Figure 3C) and NO production (Figure 3D) as well as treated with iNOS inhibitor 1400W.

OxLDL rapidly elevates ROS and then downstream events are activated involving p38 mitogen-activated protein kinase (MAPKs) [20]. There is abundant evidence for activation of the MAP kinases system by NADPH oxidases [21]. Then causing NF-κB activation and enabling nuclear translocation [22]. Numerous transcription factors are considered to redox control, including NF-κB, Nrf2, AP-1, HIF-1, and P53. NF-κB acts a critical role in the regulation of pro-inflammatory genes expression and is considered the prototypical transcription factor to redox control [23]. Our results showed that baicalein could inhibit NF-κB activation and downstream pro-inflammatory response including increased levels of COX-2 (Figure 4).

Baicalein protects against oxidative stress-induced oxidative injuries by modulation of SOD and catalase activity [24, 25]. This study indicated that the ability of baicalein to quench oxidative stress is conveyed through the suppression of ROS generation. The molecular mechanisms of baicalein suppress ROS generation is probably because baicalein belongs to phenolic antioxidants (PhOH) able to transfer electron free radicals by donation of a hydrogen atom to radicals. Unlike the hydroxyl (.OH) and superoxide (O-2) radicals, which are highly active due to a very short half-life, the phenoxy radical intermediates are relatively stable so they do not initiate further radical reactions and thereby stop the radical reaction chain [26]. The effect of baicalein resulted from decreased ROS generation subsequently attenuated oxLDL-impaired superoxide dismutase (SOD) and suppressed ROS-induced intracellular signaling pathways leading to reverse gene transcription and protein synthesis. Baicalein also maintained oxLDL-impaired mitochondria membrane potential (Figure 5), thereby prevented the release of mitochondrial protein cytochrome c, a molecule required for the activation of caspase-3 that executes the cell death program.

Flavonoids are likely nontoxic for long term therapies. Scutellaria baicalensis has been shown to have almost no or very low toxicity to animals and humans, and so far baicalein at the doses have been shown to have no or very little toxicity to normal cells [26]. The concentrations of baicalein (2.5-20 μM) required suppressing the oxLDL-induced endothelial dysfunction in our study were similar to those reported to inhibit other responses, such as inhibit the invasion of MDA-MB-231 human breast cancer cells (10-40 μM) [27]. In humans, it is unclear how much the circulating blood level would be elevated by a single dose of baicalein, since the pharmacokinetics of the components of baicalein have not been completely established. It also is unknown whether prolonged use of baicalein would lead to chronic accumulation of some of the components in different tissues.

In summary, we have demonstrated that baicalein mitigated oxidative stress-related responses by regulating NO signaling. Our results provide insight into some of the mechanisms by which baicalein inhibits endothelial oxidative damage.

## MATERIALS AND METHODS

### Cell culture and reagents

Human umbilical vein endothelial cells (HUVECs) were obtained from ATCC. HUVECs were cultured with M199 basal medium supplemented with low-serum growth supplement and penicillin (50 IU/ml)-streptomycin (50 μg/ml). Trypsin-EDTA was used to passage cells. M199 and trypsin-EDTA were obtained from Gibco (Grand Island, NY, USA). Low-serum growth supplement was purchased from Cascade (Portland, OR, USA). Additionally. DHE, L-NIO, SNP, 1400W, penicillin and streptomycin were all purchased from Sigma (St. Louis, MO, USA). Anti-β-actin, anti-eNOS, anti-iNOS, anti-cytochrome c, anti-P53, anti-p-P53, anti- nitrotyrosine, anti-Bcl-2,anti-Bax were all obtained from Santa Cruz Biotechnology (Santa Cruz, CA, USA). Baicalein was obtained from Sigma and solved in DMSO.

### Lipoprotein separation

Human plasma was obtained from the Taichung Blood Bank (Taichung, Taiwan) and LDL was isolated using sequential ultracentrifugation (= 1.019-1.063 g/ml) in KBr solution containing 30 mM EDTA, stored at 4°C in sterile, dark environment and used within 3 days as previously described. Immediately before the oxidation tests, LDL was separated from EDTA and from diffusible low molecular mass compounds by gel filtration on PD-10 Sephadex G-25 Mgel (Pharmacia) in 0.01 mol/l phosphate-buffered saline (136.9 mmol/l NaCl, 2.68 mmol/l KCl, 4 mmol/l Na2HPO4,1.76 mmol/l KH2PO4) at pH 7.4. Cu2+-modified LDL (1mg protein/ml) was prepared by exposing LDL to 10 μM CuSO4 for 16 hrs at 37°C. Protein concentration was determined by Bradford Protein Assay.

### Measurement of ROS production

The effect of baicalein on ROS production in HUVECs was determined by a fluorometric assay using dihydroethidium (DHE). Confluent HUVECs (10^4^ cells/well) in 96-well plates were preincubated with various concentrations of baicalein for 2 hrs; After the removal of medium from wells, cells were incubated with 10 μM DHE for 1 hr. oxLDL was then added to the medium in the absence or presence of baicalein for 2 hrs. The fluorescence intensity was measured with a fluorescence microplate reader (Labsystem, CA) calibtated for exciation at 485 nm and emission at 538 nm. The percentage increase in fluorescence per well was calculated by the formula [(Ft2-Ft0)/Ft0] X 100, where Ft2 is the fluorescence at 2 hrs of oxLDL exposure and Ft0 is the fluorescence at 0 min of oxLDL exposure.

### Immunoblotting

To determine whether baicalein could ameliorate the oxLDL-induced protein. HUVECs were grown to confluence, pretreated with baicalein for 2 hrs and then stimulated with oxLDL for 24 hrs. At the end of stimulation, cells were washed, scraped from dishes, and lysed in RIPA buffer (in mM: HEPES 20, MgCl2 1.5, EDTA 2, EGTA 5, dithiothreitol 0.1, phenylmethylsulfonyl fluoride 0.1, pH 7.5). Proteins (30 μg) were separated by electrophoresis on SDS-polyacrylamide gel. After the protein had been transferred to polyvinylidene difluoride membrane (Millipore, Bedford, MA), the blots was incubated with blocking buffer (1X PBS and 5% nonfat dry milk) for 1 hr at room temperature and then probed with primary antibodies overnight at 4°C, followed by incubation with horseradish peroxidase-conjugated secondary antibody (1:5000) for 1 hr. To control equal loading of total protein in all lanes, blots were stained with mouse anti-β-actin antibody at a 1:50000 dilution. The bound immunoproteins were detected by an enhancer chemiluminescent assay (ECL; Amersham, Berkshire, UK). The intensities were quantified by densitometric analysis (Digital Protein DNA Imagineware, Huntington Station, NY).

### Nuclear protein extraction

Cells grown to 80% confluency and subjected to various treatments were subsequently washed with ice-cold PBS and it was prepared for nuclear protein extraction. Cells grown on 10-cm dish were gently scraped with 3 ml ice-cold PBS and it were centrifuged at 1,000x g for 10 min at 4°C. After carefully aspirating the supernatant, cells were resuspended with 200 μl ice-cold BUFFER-I (10 mM Hepes (pH 8.0), 1.5 mM MgCl2, 10 mM KCl, 1 mM dithiothreitol, and proteinase inhibitor cocktail (Roche Molecular Biochemicals) and incubated for 15 min on ice to allow cells to swell, followed by adding 20 ll IGEPAL-CA630. After vigorously vortexing for 10 s and centrifuging at 16,000 g for 5 min at 4°C, the supernatant (cytoplasmic fraction) were carefully aspirated and the pellet were resuspended with ice-cold BUFFER-II (20 mM Hepes (pH 8.0), 1.5 mM MgCl2, 25% glycerol, 420 mM NaCl, 0.2 mM EDTA, 1 mM dithiothreitol and proteinase inhibitor cocktail (Roche Molecular Biochemicals)) and vigorously vortex. After vortexing, the suspension was placed on ice for 30 min before centrifuging at 16,000x g for 15 min at 4°C. The supernatants (nuclear extracts) were stored aliquots at −80°C. Protein concentration of the supernatants was determined by the colorimetric assay (Bradford).

### Nitrite (NO2-) accumulation

NO2- accumulation was used as an indicator of NO production in the medium and was assayed by Gries reagent. Briefly, 100 μl of Gries reagent (1% sulfanilamide-0.1% naphthylethylene diamine dihydrochloride-2.5% H3PO4) (Sigma, St. 12 Louis, MO) was added to 100 μl of each supernatant in triplicate wells of 96-well plates. The plates were read in a microplate reader (Molecular Devices, Palo Alto, CA, USA) at 550 nm against a standard curve of NaNO2 in culture medium.

### Measurement of mitochondria membrane potential

The lipophilic cationic probe fluorochrome 5,58,6,68-tetraethylbenzimidazol- carbocyanine iodide (JC-1) was used to explore the effect baicalein on the mitochondria membrane potential (ΔΨm). JC-1 exists either as a green fluorescent monomer at depolarized membrane potential or as a red fluorescent J-aggregate at hyperpolarized membrane potential. JC-1 exhibits potential-dependent accumulation in mitochondria, as indicated by the fluorescence emission shift from 530 to 590 nm. After treating cell with oxLDL for 24 hrs in the presence or absence various concentrations of baicalein, cells were rinsed with M199, and JC-1 (5μM) was loaded. After 20 min of incubation at 37°C, cell were examined under a fluorescent microscope. Determination of the ΔΨm was carried out using a FACScan flow cytometer.

### Isolation of cytosolic fraction for cytochrome c analysis

After treating cells with oxLDL in the presence and absence of natural products, the cells were collected and lysed with lysis buffer (20mmol/L HEPES/NaOH, pH 7.5, 250 mmol/L sucrose, 10 mmol/L KCl, 1.5 mmol/L MgCl2, 2 mmol/L EDTA, 5 mmol/L EGTA, 1 mmol/L DTT, protease inhibitor cocktail) for 20 min on ice. The samples were homogenized 30 strockes by glass Dounce and pestle. The homogenates were then centrifuged at 500x g to remove unbroken cells and nuclei. Supernatant were centrifuged at 17000x g for 30 min to isolate mitochondria fraction. Supernatant was cytoslic extraction and pellet was mitochondria fraction lysed by RIPA buffer. Cytosol and mitochondria protein were resolved by SDS-polyacryamide gel electrophoresis.

### Measurement of active caspase-3

Cells were pretreated with baicalein for 2 hrs and then stimulated with oxLDL for 24 hrs. At the end of stimulation, cells were harvested and incubated with commercial fluorescein active caspase kit (Mountain View, CA) for 45 min at room temperature. The level of active caspase-3 was detected by flow cytometry and fluorescence microscope.

### Determination of cytotoxicity and induce of apoptosis

Cells were first incubated with baicalein for 2 hrs and then stimulated with oxidized LDL for 24 hrs. At the end of stimulation, mitochondrial dehydrogenase activity, which can be used as an index of cell viability, was assessed using the MTT (Sigma) assay. Plasma membrane integrity was assessed by measuring lactate dehydrogenase (LDH) release using an LDH diagnostic kit (Biovision) according to the manufacture's instructions. Apoptotic cells were assessed by a terminal deoxynucleotidyl transferase-mediated dUTP nick end-labeling (TUNEL) assay (Roche) under a fluorescence microscope or in a flowcytometer.

### Measurement of active caspase-3

HUVECs were pretreated with baicalein for 2 hrs and then stimulated with oxLDL for 24 hr. In some cases. The activity of caspase-3 was also measured by an EnzChek caspase-3 assay kit according to the manufacturer's instructions (Molecular Porbes Inc, Eugene, OR). After being lysed by repeated freeze-thaw cycles. Equal amounts of protein (50 μg) were added to the reaction buffer containing 5 mM of caspase-3 substrate Z-DEVD-R110 and the mixture was incubated at room temperature for 30 min. The fluorescence generated from cleavage of the substrate by caspse-3 was monitored with a fluorescence microplate reader (Labsystem, CA) calibrated for excitation at 496 nm and for emission at 520 nm.

### Statistical analyses

All experiments were repeated 3 times, and one of these results are provided. Results are expressed as mean±SEM. Differences between the groups were analyzed using one-way ANOVA followed by the Student's t test. A *P*-value < 0.05 was considered statistically significant.
